# Research progress on oral glucagon-like peptide-1 receptor agonists in the treatment of diabetes mellitus type 2

**DOI:** 10.3389/fmolb.2025.1729904

**Published:** 2026-01-07

**Authors:** Qian Shao, Juan Xiong, Jing Wu, Jingxin Mao, Qing Hu

**Affiliations:** 1 Chongqing Municipal Key Laboratory for High Active Traditional Chinese Drug Delivery System, Chongqing Medical and Pharmaceutical College, Chongqing, China; 2 Department of Science and Education, The First Affiliated Hospital of Chongqing Medical and Pharmaceutical College, Chongqing, China

**Keywords:** glucagon-like peptide-1 receptor agonist, multi-targetmechanism, pancreatic β-cell function, research progress, type 2 diabetes mellitus

## Abstract

**Objective:**

In view of the high incidence of type 2 diabetes mellitus (T2DM) and the high prevalence of multi-organ complications, as well as the issues that traditional hypoglycemic drugs are prone to causing weight gain and the molecular targets and signaling pathways of classic drugs such as metformin have not been systematically clarified, this study aims to systematically analyze the mechanism of action and clinical value of glucagon-like peptide-1 receptor agonists (GLP-1RAs), and It further clarifies key signaling pathways including adenosine monophosphate-activated protein kinase (AMPK), phosphatidylinositol 3-kinase (PI3K)-protein kinase B (Akt), cyclic adenosine monophosphate (cAMP)-protein kinase A (PKA), and interleukin-6 (IL-6)/signal transducer and activator of transcription 3 (STAT3) cytokine pathways, prkviding theoretical support for precision interventions in T2DM.

**Methods:**

The latest domestic and international multi-omics research data, cell/animal functional experiment results, and clinical evidence were systematically integrated to analyze the structural modification strategies and glucose concentration-dependent mechanism of action of GLP-1RAs. Emphasis was placed on dissecting their regulatory pathways for insulin/glucagon secretion, as well as key receptor-related networks.

**Result:**

Glucagon-like peptide-1 receptor agonist (GIP-1RA), when modified at specific amino acid positions, becomes resistant to dipeptidyl peptidase 4 (DPP-4) degradation. It activates the Gs/cAMP/PKA/exchange protein activated by cAMP (EPAC) signaling axis to promote insulin release in a glucose concentration-dependent manner, while suppressing glucagon secretion through Gi/cAMP downregulation and insulin synergistic effects. Additionally, it induces transient IL-6 release in monocytes, enhancing adipose tissue brownification and thermogenesis via the IL-6/STAT3 pathway. This mechanism protects pancreatic β-cells by preventing apoptosis and promoting proliferation, while improving insulin resistance in adipose, hepatic, and skeletal muscle tissues. The compound also exhibits dual effects of weight loss and hepatoprotective (miRNA-regulated lipid metabolism) and nephroprotective (sodium excretion and anti-inflammatory) actions. Key regulatory targets include AMPK, PI3K-Akt, cAMP-PKA, and IL-6/STAT3.

**Conclusion:**

GLP-1RAs overcome the limitations of endogenous GLP-1 and traditional hypoglycemic drugs, providing a new strategy for the comprehensive treatment of T2DM featuring “hypoglycemia-organ protection-weight loss”. The mechanisms and pathway networks analyzed in this study lay a foundation for the precise intervention of T2DM and rational clinical drug use.

## 1 Introduction

Type 2 diabetes mellitus (T2DM) is a chronic metabolic disease with a rapidly increasing global incidence, characterized by chronic hyperglycemia (Galicia-Garcia et al., 2020). According to the 2022 International Diabetes Federation statistics, there are approximately 536 million people with diabetes worldwide, and this number is projected to reach 783 million by 2045 (Leite, 2025). As the disease progresses, patients are prone to develop a series of serious complications such as diabetic nephropathy and retinopathy. These complications not only increase the medical burden on patients but also significantly reduce their quality of life. In addition, patients with T2DM face a higher risk of cardiovascular disease, with a prevalence 1.5 to 3 times that of the general population (Einarson et al., 2018). Therefore, there is an urgent clinical need to develop both safe and effective therapeutic strategies. However, current mainstream T2DM treatments—such as insulin and sulfonylureas—are commonly accompanied by weight gain (Chaudhury et al., 2017), making it difficult to meet patients demand for comprehensive metabolic management. Against this backdrop, glucagon-like peptide-1 (GLP-1) has attracted widespread attention. GLP-1 is a multifunctional peptide hormone secreted by intestinal L-cells under the regulation of nutrient intake, neural and endocrine signals (Lim and Brubaker, 2006). It not only modulates glucose homeostasis by promoting insulin synthesis and secretion, suppressing glucagon release, delaying gastric emptying, and reducing food intake, but also stimulates proliferation and neogenesis of pancreatic β-cells while inhibiting their apoptosis, thereby offering a key therapeutic target for T2DM. Glucagon-like peptide-1 receptor agonists (GLP-1RAs), developed based on GLP-1, represent a novel class of glucose-lowering drugs that have demonstrated notable advantages in overweight or obese patients with T2DM (Gourdy et al., 2023). In addition to reliable glycemic control, they effectively reduce body weight and show potential for cardiovascular protection. To date, nine glucagon-like peptide-1 receptor agonist (GLP-1RA) formulations have been approved for clinical use in China (Yao et al., 2024). Although all these agents lower blood glucose by activating the endogenous GLP-1 receptor, differences in their molecular structures and amino-acid homology to native GLP-1 result in considerable heterogeneity in pharmacokinetic profiles and clinical efficacy. In addition, GLP-1RAs have been shown to ameliorate central neurodegeneration (Monti et al., 2022) and exhibit promising therapeutic effects in Alzheimer’s disease, vascular dementia, and Parkinson’s disease. Consequently, expert consensus now recommends GLP-1RAs as an important therapeutic option after metformin. Although GLP-1RAs have become a cornerstone of T2DM management, and their glucose-dependent glucose-lowering efficacy, weight-reducing action, and organ-protective potential are widely recognized, current research remains largely confined to single-agent explorations or short-term outcome evaluations. A systematic integration of their multi-target networks is still lacking, and comparative studies on how distinct structural-modification strategies enhance enzymatic stability or refine tissue selectivity remain insufficient. Moreover, the precise molecular mechanisms through which GLP-1RAs protect the liver, kidney, and central nervous system have so far been demonstrated only in animal models (Grieco et al., 2019), and validation in human clinical samples is still lacking. Likewise, real-world data that compares the efficacy, long-term safety, and health-economic profiles of long-versus short-acting GLP-1RA formulations remain scarce. Driven by these gaps, the present review integrates the latest omics datasets and functional experimental findings to systematically chart the research-and-development rationale, mechanisms of action, and clinical value of GLP-1RAs, thereby providing a theoretical framework for precision intervention and rational use in T2DM and delineating future research directions in this field.

## 2 Overview of diabetes

### 2.1 Pathogenesis of diabetes

The pathogenesis of diabetes is multidimensional and complex, revolving around two main subtypes, type 1 diabetes mellitus (T1DM) and T2DM (Zaccardi et al., 2016). Although their mechanisms differ markedly, both ultimately disrupt glucose homeostasis. T1DM arises when genetic susceptibility and environmental triggers—such as viral infections—ignite an autoimmune response (Ilonen et al., 2019) that destroys pancreatic β-cells, leading to an absolute insulin deficit and loss of glycaemic control (Katsarou et al., 2017). Accounting for more than 90% of all diabetes cases, T2DM is driven by core defects of insulin resistance and progressive β-cell failure (Author anonymous, 2017), peripheral tissues (muscle, adipose, liver) become less sensitive to insulin, impeding insulin-signalling pathways and causing defective glucose uptake and utilization (Mu et al., 2019). Meanwhile, pancreatic β-cells fail to compensate adequately, their proliferative capacity declines, and apoptosis increases as the disease advances, resulting in a relative insulin deficiency that perpetuates a “resistance–under-secretion” vicious cycle (Eguchi et al., 2021). In addition, factors such as intestinal dysbiosis (e.g., altered microbial metabolites like short-chain fatty acids), chronic low-grade inflammation (e.g., adipokine imbalance), and genetic polymorphisms (e.g., TCF7L2 variants) further modulate metabolic and immune pathways, contributing to the development of diabetes and together forming a complex pathophysiological network.

### 2.2 Social impact of diabetes

Accelerated population ageing, rapid urbanisation, and changing lifestyles have led to a continuous rise in the incidence of diabetes, obesity and other chronic non-communicable metabolic diseases, posing a major public-health threat to the nation (Habib and Saha, 2010). In 2020, the number of people with diabetes in China reached 260.4 million, with older adults accounting for 30% of this figure and T2DM being the predominant form (Zhang Z. et al., 2024), nevertheless, clinical data show that only about 49.2% of patients with T2DM achieve the glycaemic target of glycated hemoglobin A1c (HbA1c) < 7.0% (Qaseem et al., 2018), chronic hyperglycaemia markedly increases the risk of chronic complications such as diabetic peripheral neuropathy—which principally affects sensory and motor nerves and seriously disrupts daily life—and also damages the eyes, heart, kidneys and other vital organs, precipitates acute metabolic crises, and remains a leading cause of cardiovascular disease, blindness and renal failure (Lotfy et al., 2017). From a global perspective, the International Diabetes Federation’s IDF Diabetes Atlas 2021 identifies diabetes as the fastest-growing global health emergency of the 21_st_ century (Van Netten et al., 2024). These data underscore the enormous unmet clinical need for diabetes and obesity management and highlight the critical practical and therapeutic value of developing safe, effective prevention and treatment strategies.

## 3 Overview of GLP-1

### 3.1 GLP-1 synthesis and actions

GLP-1 is a peptide hormone synthesized and secreted by intestinal L-cells (Chun and Butts, 2020), its release is jointly regulated by nutrient intake, neural inputs, and endocrine signals, endowing it with multiple biological functions including glycaemic control (Sandoval and D'Alessio, 2015). The hormone exerts its effects through the specific GLP-1R, which is predominantly localized to pancreatic islet β-cells (Mei et al., 2002). Upon binding to and activating GLP-1R, GLP-1 employs a dual-control mechanism to regulate glucose metabolism, it markedly stimulates pancreatic β-cells to synthesize and secrete insulin, thereby enhancing whole-body glucose uptake and utilization, while simultaneously suppressing glucagon secretion to curtail endogenous glucose production (Pabreja et al., 2014). Through this synergistic action GLP-1 efficiently restores glucose homeostasis and achieves a sustained hypoglycaemic effect, providing a pivotal target for diabetes-related research.

### 3.2 Structure and function of GLP-1R

GLP-1R belongs to the class B family of g-protein-coupled receptors and is widely expressed in multiple organs, including the brain, heart, pancreas, and gastrointestinal tract. The gene is located on chromosome 6 (6p21.2), spans 42.5 kb, and encodes a protein of 463 amino acids (Brubaker and Drucker, 2002). The receptor comprises a typical extracellular domain (ECD) and transmembrane domain (TMD). The ECD (residues 58–135) contains a flexible α-helix, two antiparallel β-sheets, and six conserved cysteines that confer specific ligand recognition (Hohenester and Engel, 2002). Upon binding to the receptor, the ligand’s C-terminal helix first docks with the ECD, and its N-terminal segment then engages the TMD to activate the receptor (Changeux and Christopoulos, 2016). This activation triggers the dissociation of the G-protein α-subunit from the β/γ complex and initiates intracellular signalling (Changeux and Christopoulos, 2016). GLP-1 is an incretin hormone encoded by the pro-glucagon gene on chromosome 2 (six exons and five introns, with the coding region in exon 4) and secreted by intestinal L-cells. Its release is triggered by intraluminal glucose and other nutrients, but the peptide has a half-life of only 1–2 min because it is rapidly inactivated by DPP-4, thus, its biological effects are exerted primarily through binding to GLP-1R (Zhao et al., 2016). In the pancreas, binding of GLP-1 to its receptor activates adenylyl cyclase (AC), raising intracellular cyclic adenosine monophosphate (cAMP) levels and thereby stimulating both protein kinase A (PKA) and Epac2 pathways, PKA promotes expression of proinsulin genes and insulin synthesis by modulating ion channels (closing K^+^ channels and opening voltage-dependent Ca^2+^ channels), while Epac2 facilitates fusion of insulin granules with the plasma membrane and the release of secretory vesicles (Lang, 1999). Meanwhile, the phosphatidylinositol 3-kinase (PI3K)/protein kinase B (Akt) andmitogen-zctivated protein kinase (MAPK) pathways—including cAMP- and cAMP-GEF-mediated cross-talk—are activated to promote β-cell proliferation and differentiation while suppressing apoptosis (Hou et al., 2009). cAMP/Epac2 also upregulates cAMP response element-binding protein and anti-apoptotic factors such as B-cell cLL/lymphoma 2 (Bcl-2) and B-cell lymphoma-extra large (Bcl-XL). Within the gastrointestinal tract GLP-1 slows motility and gastric emptying, restrains food intake and augments satiety, thereby reducing body weight, and it blunts post-prandial chylomicron secretion to lower triglycerides (Wang et al., 2023). GLP-1R agonists further attenuate hepatic steatosis and inflammation in models of non-alcoholic steatohepatitis (NASH), alleviating hepatocyte injury (Nevola et al., 2023).

### 3.3 Distribution and function of GLP-1R

GLP-1R belongs to the class B G-protein-coupled receptor family (Donnelly, 2012) and is widely distributed across multiple tissues and organs, with the highest expression in the pancreatic islets and gastrointestinal tract, it is also present in the central nervous system and cardiovascular system, providing the structural basis for its pleiotropic regulatory effects (Wen et al., 2021). Within the pancreatic islets, GLP-1R is predominantly localized to β-cells and is expressed at lower levels in α-cells (Tong et al., 2025). Upon binding of GLP-1 to GLP-1R on the β-cell surface, Gs protein is rapidly activated, causing intracellular cAMP levels to rise and triggering a downstream signalling cascade that culminates in multiple physiological effects (Lymperopoulos et al., 2025), insulin release is accelerated, while β-cell apoptosis is suppressed and their proliferation and differentiation are enhanced, thereby preserving β-cell functional homeostasis. Within the gastrointestinal tract, activation of GLP-1R slows gastric emptying, suppresses gastric acid and pepsin secretion, and reduces intestinal motility, thereby decreasing food intake and nutrient absorption and effectively blunting post-prandial glucose excursions (Farré and Tack, 2013). In the central nervous system, GLP-1R is localized to areas critical for appetite regulation and energy balance, notably, in the hypothalamus its activation promotes satiety, curbs appetite, and lowers food intake (Drucker, 2022). In addition, GLP-1R expression in the cardiovascular system confers direct cardioprotection on GLP-1, manifesting as reduced myocardial ischemia–reperfusion injury and improved cardiac function, thereby further broadening the physiological regulatory value of GLP-1R (Saraiva and Sposito, 2014).

### 3.4 Limitations of GLP-1 in diabetes therapy and the discovery of GLP-1RA

GLP-1 is a key incretin and a master signal for glucose homeostasis (Baggio and Drucker, 2007), its biological effects require specific binding to and activation of GLP-1R on target cells (Zou et al., 2025). This engagement modulates metabolism in two ways, it stimulates β-cell insulin biosynthesis and secretion while suppressing α-cell glucagon release, achieving bidirectional glycaemic control (Quesada et al., 2008), and it slows gastric emptying to blunt post-prandial glucose peaks and stabilise overall glycaemia (Meloni et al., 2013). However, native GLP-1 has a severe pharmacokinetic flaw that limits its direct use—its circulating half-life is only 2 min, too short to sustain meaningful biological activity (Aaboe et al., 2008). The culprit is rapid degradation by DPP-4, a serine protease that selectively cleaves the Ala^2^–His^1^ peptide bond at the N-terminus of GLP-1 (Zhang M. et al., 2024; Mulvihill and Drucker, 2014), inducing a conformational change that abolishes receptor binding and leads to prompt inactivation and clearance. To circumvent this hurdle, drug-discovery efforts have focused on GLP-1RAs engineered for proteolytic resistance. The dominant strategy is site-specific amino-acid substitution, replacing Ala^8^ with glycine or α-aminoisobutyric acid markedly reduces DPP-4 recognition (Lam et al., 2025), prolongs residence time, and extends duration of action, providing a foundation for long-acting pharmacotherapy of T2DM and obesity.

## 4 Clinical applications of GLP-1RA

### 4.1 Introduction to GLP-1RA

GLP-1RAs lower blood glucose by activating the GLP-1 receptor and potentiating glucose-dependent insulin secretion (Zhao et al., 2021). The first agent of this class, exenatide, was approved by the U.S. food and drug administration for patients with T2DM in 2005. Besides glycaemic control, it also confers beneficial effects on body-weight reduction, blood-pressure lowering, and renal and hepatic protection. Currently, liraglutide, semaglutide, dulaglutide and polyethylene-glycol loxenatide are available in China. Since liraglutide was included in the National Reimbursement Drug List in 2018, the use of GLP-1-based therapies has entered a phase of explosive growth (Turnock et al., 2025), and in 2022 their market size exceeded that of insulin for the first time.

### 4.2 Clinical pharmacological effects of GLP-1RA

Glycaemic control is achieved through glucose-dependent activation of the GLP-1 receptor, which augments insulin secretion (Hedeskov, 1980) by enhancing voltage-gated Ca^2+^ influx and calmodulin activation via endoplasmic-reticulum Ca^2+^ release, while simultaneously suppressing glucagon secretion (Ramracheya et al., 2010). As plasma glucose returns to normal, the stimulatory effect wanes automatically, markedly lowering the risk of hypoglycaemia. For weight reduction, GLP-1RAs act on central appetite circuits to inhibit hunger, delay gastric emptying, and dampen gastric motility, thereby increasing satiety and decreasing food intake (Jalleh et al., 2024). Clinical studies show that short-term liraglutide reduces central food-cue responses in T2DM, although long-term effects require further validation (Anyiam et al., 2024). Organ protection is partly mediated by miRNA regulation in the liver (Schueller et al., 2018). GLP-1RAs downregulate hepatic miR-34a/21 and upregulate miR-200b/c, decreasing intrahepatic lipid accumulation and steatosis and providing a rationale for treating NASH, although protection appears confined to livers with simple steatosis (Petrovic et al., 2023). Renal protection operates through multiple mechanisms, including inhibition of proximal-tubular Na^+^/H^+^ exchange to promote natriuresis and lower blood pressure, activation of PKA and inhibition of protein kinase C (PKC) can attenuate renal injury, meanwhile, upregulation of atrial natriuretic peptide activates the protein kinase G (PKG) pathway, which in turn exerts anti-inflammatory and antioxidant effects and improves renal arterial perfusion (Aperia et al., 1994). It should be noted that because GLP-1RAs lower glucose by potentiating insulin secretion, they are not suitable for type 1 diabetes and cannot replace insulin therapy (Nauck et al., 2022).

### 4.3 Mechanisms of GLP-1RA in the treatment of T2DM

#### 4.3.1 Specific signaling pathways of GLP-1RA in T2DM therapy

**FIGURE 1:**
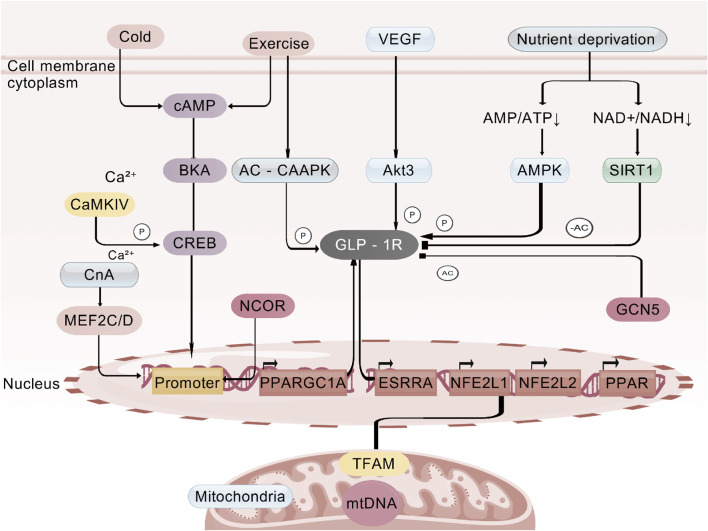
cAMP-PKA (core glucose-lowering) pathway.

**FIGURE 2:**
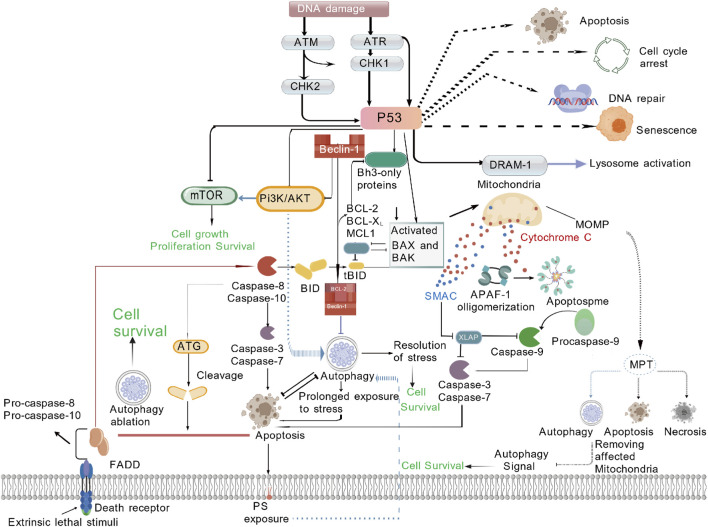
PI3K-Akt (β-cell protection and peripheral glucose regulation) pathway.

**FIGURE 3:**
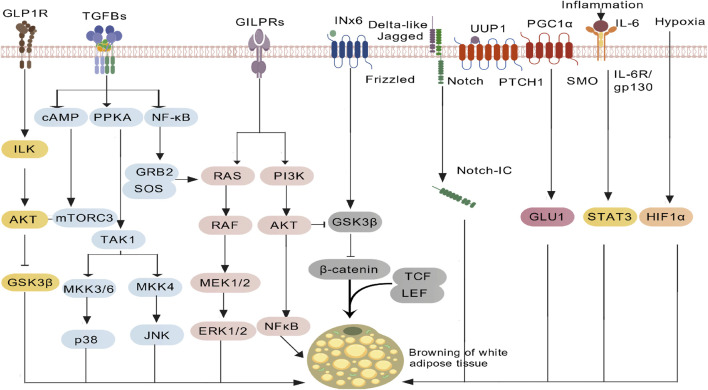
AMPK (β-cell proliferation) pathway.

**FIGURE 4:**
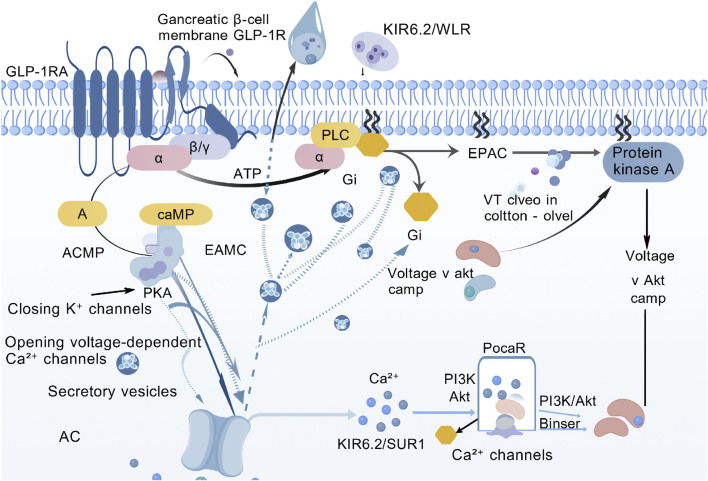
IL-6/STAT3 (adipose tissue browning) pathway.

Rather than relying on a single pathway, GLP-1RAs coordinately engage four core signalling axes—cAMP-PKA, PI3K-Akt,interleukin-6 (IL-6)/signal transducer and activator of transcription 3 (STAT3) and adenosine monophosphate-activated protein kinase (AMPK)—together with central and peripheral regulatory networks to achieve glycaemic control (Table 1). cAMP-PKA axis, After GLP-1RA binds GLP-1R, Gs-protein-coupled AC is activated, raising intracellular cAMP. cAMP in turn stimulates PKA and exchange protein activated by cAMP (EPAC), closes KIR6.2/SUR1 K^+^ channels, opens voltage-dependent Ca^2+^ channels (VDCCs) and triggers Ca^2+^ influx, promoting insulin-granule exocytosis. This sequence is strictly glucose-dependent—engaged only when plasma glucose is elevated—so hypoglycaemia risk is minimal (Figure 1). PI3K-Akt axis, Within β-cells the PI3K-Akt cascade downregulates pro-apoptotic bcl-2 associated death rromoter (Bad) and bcl-2 associated x protein (Bax), upregulates Bcl-2, boosts antioxidant enzymes (superoxide dismutase (SOD), catalase (CAT)) and suppresses tumor necrosis factor-α (TNF-α)/IL-6-mediated inflammation, delivering dual “anti-apoptotic + pro-proliferative” protection. In peripheral tissues the same pathway drives glucose transporter 4 (GLUT4) translocation to the membrane and inhibits hepatic glucose output (PEPCK/G6Pase), ameliorating insulin resistance (Figure 2). AMPK axis, GLP-1RAs elevate adiponectin, activating AMPK, decreasing free fatty acid (FFA) release, restraining hepatic lipid deposition (down-regulating miR-34a/21 and up-regulating miR-200b/c), and increasing GLUT4 expression in skeletal muscle, thereby augmenting glucose uptake and sustaining multi-organ metabolic homeostasis (Figure 3). In terms of cytokine signaling mechanisms, IL-6-mediated STAT3 activation is crucial for the regulation of adipocyte differentiation and function, in preadipocytes, activated STAT3 phosphorylates and translocates into the nucleus to regulate the expression of thermogenesis-related genes, promoting browning of white adipose tissue and enhancing thermogenic efficiency; the IL-6/STAT3 signaling also upregulates thermogenic genes such as uncoupling protein 1 (UCP1) and peroxisome proliferator-activated receptor γ coactivator 1α (PGC-1α) to increase adipose energy expenditure, improve glucose metabolism, thereby synergistically contributing to the anti-diabetic and metabolic improvement effects of GLP-1RAs (Figure 4).

**TABLE 1:** Overview of pathways of GLP-1RA for the treatment of diabetes.

Pathway name	Core biological function	Core mechanism of action	Key molecules/Targets	Literature support
cAMP-PKA	Glucose-dependent blood glucose lowering (avoiding hypoglycemia)	GLP-1RA→GLP-1R→Gs→AC↑→cAMP↑→PKA/EPAC→closing KIR6.2/SUR1, opening VDCC→Ca^2+^↑→insulin exocytosis	GLP-1R, Gs, AC, cAMP, PKA,EPAC, KIR6.2/SUR1, VDCC	Petrovic et al. (2023), Mei et al. (2002), Tomlinson et al. (2022), Warwar et al. (2006), Jones et al. (2018)
PI3K-Akt	β-cell protection and improving insulin resistance	Anti-apoptosis, ↓Bad/Bax, ↑Bcl-2, antioxidation, anti-inflammation, Pro-proliferation, ↑CyclinD1/PCNA, Peripheral tissues, ↑adiponectin, ↓hepatic glucose output, ↑GLUT4	PI3K, Akt, Bcl-2, Bad/Bax, SOD, CAT, CyclinD1, PCNA, GLUT4,a diponectin, PEPCK, G6Pase	Tengholm and Gylfe (2017), He et al. (2025), Wang and Wang (2017), Sesti (2006), Zhang X et al. (2025)
AMPK	β-cell proliferation and multi-organ metabolic homeostasis	Activation of AMPK→promote β-cell proliferation, Adiponectin→↓FFA, Hepatic miR→reduce lipid accumulation, Skeletal muscle→↑GLUT4	AMPK, adiponectin, FFA,miR-34a/21, miR-200b/c, GLUT4	Jalleh et al. (2024), Wang et al. (2023), Zhou et al., 2025
IL-6/STAT3	Brownification of adipose tissue + heat production + glucose utilization	GLP-1RA→hyaline leukocyte GLP-1R→IL-6↑→axunge IL-6R/gp130→STAT3→UCP1/PGC-1α↑	GLP-1R,IL-6,IL6R/gp130, STAT3,UCP1,PGC-1α	Essaouiba (2020), Zhang X et al. (2025), Xu et al. (2009)

#### 4.3.2 Accelerating the release of insulin

GLP-1RA specifically bind to GLP-1R on the surface of pancreatic β-cell membranes, initiating a cascade signaling pathway mediated by Gs protein-coupled receptors, thereby achieving precise regulation of insulin secretion (Yang et al., 2014). Its core pathway is as follows, After receptor binding, Gs proteins are activated, driving conformational changes and activation of AC. AC then catalyzes the conversion of intracellular Adenosine Triphosphate (ATP) to cAMP, leading to a significant increase in cAMP levels. The elevated cAMP further activates PKA and EPAC (Mei et al., 2002). These two molecules synergistically regulate the opening of membrane calcium channels, accelerating extracellular calcium ion influx and promoting the migration, anchoring, and membrane fusion of insulin-containing secretory vesicles, ultimately achieving extracellular insulin release. In addition, GLP-1RA can inhibit the ion transport function of inwardly rectifying potassium channels (KIR6.2/SUR1) in pancreatic β-cells (Tomlinson et al., 2022). This reduces potassium ion efflux, triggering membrane depolarization, which further promotes the opening of VDCC and enhances the calcium influx effect, forming a “dual regulatory pathway” for insulin secretion (Warwar et al., 2006). Compared with traditional sulfonylurea secretagogues, the secretagogue effect of GLP-1RA is strictly glucose concentration-dependent (Ashik, 2023). When blood glucose exceeds the physiological threshold, the binding efficiency of GLP-1RA to GLP-1R and the activation intensity of downstream signals are significantly enhanced, efficiently promoting insulin release to lower blood glucose (Jones et al., 2018). When blood glucose returns to the normal range, the activation efficiency of this signaling pathway decreases with the decline in blood glucose, and insulin secretion is correspondingly reduced (Henquin, 2000), thereby reducing the clinical risk of hypoglycemia at the molecular mechanism level.

#### 4.3.3 Inhibiting glucagon release

Pancreatic α-cells, as the main secretory source of glucagon, express functional GLP-1R on their cell membrane surfaces, providing an important target of action for GLP-1RA (Shilleh et al., 2024). After GLP-1RA specifically binds to GLP-1R on α-cells, it initiates a Gi protein-mediated inhibitory signaling pathway (Ma et al., 2021), by activating Gi protein subtypes, it directly inhibits the catalytic activity of AC, blocks the conversion of intracellular ATP to cAMP, and leads to a significant decrease in cAMP levels in α-cells, since cAMP is a core second messenger for glucagon synthesis and secretion, the reduction in its level directly inhibits the extracellular release of glucagon granules (Tengholm and Gylfe, 2017). Meanwhile, GLP-1RA also regulates α-cells through an “indirect synergistic pathway” (He et al., 2025), it promotes insulin secretion from pancreatic β-cells, and this insulin can bind to insulin receptors on the α-cell membrane surface, activate the downstream PI3K/Akt signaling axis, and further inhibit glucagon secretion. This dual regulatory mode of “direct inhibition + indirect synergy” forms a difference in signaling pathways from the Gs protein-dependent activation effect of GLP-1RA in β-cells, and together they constitute the molecular basis for its bidirectional regulation of blood glucose—it not only lowers blood glucose by promoting insulin secretion in β-cells, but also prevents excessive blood glucose fluctuations by inhibiting glucagon release in α-cells.

#### 4.3.4 Regulating pancreatic β-cell function

Pancreatic β-cell dysfunction is a core pathological link in the progression of T2DM (Zhang X. et al., 2025), with increased β-cell apoptosis and decreased proliferation/differentiation capacity as the main causes. GLP-1RA regulates β-cell fate through multiple dimensions, in terms of anti-apoptosis, it exerts effects through three mechanisms—first, it downregulates the expression of pro-apoptotic proteins Bad and Bax via the PI3K/Akt signaling pathway (Vergès et al., 2022), while upregulating the anti-apoptotic protein Bcl-2 to maintain the balance of apoptosis-related proteins and directly inhibit apoptosis, second, it enhances the activities of SOD and CAT, reduces the production of reactive oxygen species (ROS), and alleviates oxidative stress damage (Ghosh et al., 2023), third, it inhibits the release of inflammatory mediators such as TNF-α and IL-6, and reduces β-cell damage caused by inflammation (Vallon and Thomson, 2017). In terms of promoting proliferation and differentiation, long-term hyperglycemia impairs β-cell proliferation capacity and leads to dysfunction (Wang and Wang, 2017), while GLP-1RA can activate pathways such as PI3K/Akt to promote the expression of proliferation-related genes including cyclin dopamine receptor D1 (D1) (which promotes the transition of cells from gap 1 phase (G_1_) to S phase) and proliferating cell nuclear antigen (PCNA) (which is involved in DNA replication), at the same time, it can induce the directional differentiation of pancreatic endogenous stem cells and progenitor cells into functionally mature β-cells, enrich the source of β-cells, and improve the problem of insufficient β-cell quantity.

#### 4.3.5 Improving insulin resistance

Insulin resistance is characterized by decreased insulin sensitivity and impaired glucose uptake and utilization in tissue cells as core pathological features (Sesti, 2006), and it is a key link in the progression of T2DM. GLP-1RA regulates insulin resistance through multiple targets by targeting three major metabolic tissues, adipose tissue, liver, and skeletal muscle (Petrovic et al., 2023), in adipose tissue, it regulates adipocytokine secretion, promotes adiponectin release and reduces leptin resistance, adiponectin activates the AMPK pathway to promote glucose uptake and oxidation in adipocytes, while inhibiting lipolysis to lower circulating free fatty acids, reducing their interference with the insulin receptor substrate 1(IRS-1)/PI3K/Akt insulin signaling pathway and enhancing insulin sensitivity (Notaro, 2024), in liver tissue, GLP-1RA inhibits the activity of key gluconeogenic enzymes such as phosphoenolpyruvate carboxykinase (PEPCK) and glucose-6-phosphatase (G6Pase), reduces hepatic glycogenolysis and gluconeogenesis, lowers endogenous glucose production, and at the same time regulates hepatic lipid metabolism, reduces triglyceride accumulation in hepatocytes, and alleviates the damage of lipotoxicity to hepatic insulin sensitivity (Wewer Albrechtsen et al., 2016), in skeletal muscle tissue, GLP-1RA relies on the activation of the PI3K/Akt pathway to promote the upregulation of GLUT4 expression and its membrane translocation, accelerate glucose uptake and utilization in skeletal muscle cells, and directly improve the responsiveness of skeletal muscle to insulin.

#### 4.3.6 Cytokine signaling regulation

GLP-1RAs bind to GLP-1Rs on monocytes (Kumar, 2025), and induce transient IL-6 secretion via the cyclic adenosine monophosphate-PKA-nuclear factor kappa b aignal transduction pathway (cAMP-PKA-NF-κB) axis (lasting 2–4 h, with a plasma concentration <10 pg/mL) (Romagnoli et al., 2001). Meanwhile, as confirmed by relevant studies (Yousif et al., 2021), glycoproteins secreted by dendritic cell (DC) can inhibit excessive NF-κB activation in monocytes and reduce the binding efficiency between soluble IL-6 receptor (sIL-6R) and IL-6, thereby preventing chronic IL-6 accumulation and subsequent inflammation. Circulating IL-6 binds to the IL-6 receptor/glycoprotein 130 complex on adipose tissue, activates STAT3, and promotes its nuclear translocation. This process upregulates the expression of UCP1 and PGC-1α, inducing browning of white adipose tissue and thermogenesis (Bargut et al., 2017). At the same time, it synergizes with the AMPK and PI3K-Akt pathways to enhance insulin sensitivity (Owen et al., 2000; Camaya et al., 2022).

### 4.4 The efficacy of GLP-1RA in the treatment of T2DM

GLP-1RA has significant clinical value in T2DM patients with poor glycemic control (Ren et al., 2025). In combination therapy, adding Liraglutide (subcutaneous injection once daily, half-life of 13 h) to the basis of dapagliflozin can enhance blood glucose lowering, inhibit blood glucose fluctuations, and reduce blood lipids and body mass index (James, 2008), combining metformin with polyethylene glycol loxenatide (subcutaneous injection once weekly, half-life of 5.5 days) can reduce HbA1c more significantly without adverse reactions such as hypoglycemia or weight gain, and injection site reactions are mild, making it suitable for patients who need simplified treatment regimens, adding dulaglutide (subcutaneous injection once weekly, half-life of 5.4 days) or Lixisenatide (subcutaneous injection once daily, half-life, not available) to conventional treatment can also improve islet function, lower blood glucose, and has high safety (Wu et al., 2023). In terms of monotherapy and application in special populations, semaglutide (subcutaneous injection once weekly or oral administration daily, half-life of 7 days) can lower blood glucose in elderly T2DM patients and reduce vascular endothelial damage, although high doses are more effective in lowering blood glucose, reducing blood lipids, and anti-inflammation, the risk of adverse reactions needs to be balanced, and its effects on glycemic control, weight loss, and improving islet function are better than conventional treatment alone (Toth et al., 2020),Clinical data from 8 weeks of Semaglutide treatment further confirmed that, in obese patients with T2DM, the phosphorylation level of STAT3 in subcutaneous adipose tissue increased by 38%, the proportion of beige adipocytes rose by 15%, and the level of sIL-6R in the circulation decreased by 32% under the regulation of the DC buffer system, thus preventing the activation of systemic inflammation (Xu et al., 2009). This suggests that the weight-loss effect of GLP-1RAs relies not only on the activation of the IL-6/STAT3 pathway, but also on the control of “inflammatory risk” by the DC buffer system. Benaglutide (subcutaneous injection three times daily before meals, half-life of 1.1 h), as a short-acting preparation, can significantly reduce postprandial blood glucose and has a particularly prominent weight loss effect in obese T2DM patients, but the incidence of gastrointestinal adverse reactions is relatively high, requiring initiation at a low dose, treatment with 2 mg exenatide microspheres (subcutaneous injection once weekly, half-life of 4.6 days) for 12 weeks can significantly improve islet function and alleviate β-cell dysfunction, which is the core of T2DM progression (Idris, 2019). GLP-1RA with different half-lives have their own advantages, liraglutide has good effects on controlling fasting and postprandial blood glucose, dulaglutide achieves stable blood glucose lowering, has the most significant improvement in time in range (TIR), and can delay early diabetic nephropathy, benaglutide has prominent effects on postprandial blood glucose control and weight loss, exenatide has excellent lipid regulation and low administration frequency, but gastrointestinal adverse reactions are generally common in such drugs. In terms of cost-effectiveness, insulin glargine and lixisenatide injection (subcutaneous injection once daily) can reduce treatment costs, while long-acting GLP-1RA have more significant effects on blood glucose lowering and HbA1c reduction (Table 2).

**TABLE 2:** Overview of relevant drugs for diabetes mellitus treatment.

Drug name	Administration regimen	Half-life	Core clinical advantages	Main adverse reactions	References
Exenatide	Twice daily or once weekly (subcutaneous injection)	1.3 h/4.6 days	Lipid regulation, optional short/long-acting formulations	Nausea, diarrhea (common in the initial stage)	Nevola et al. (2023), Lymperopoulos et al. (2025), Zou et al. (2025)
Liraglutide	Once daily (subcutaneous injection)	13 h	Dual control of fasting and postprandial blood glucose, improves endothelial function	Nausea, decreased appetite (relievable)	Wen et al. (2021), Tong et al. (2025), Wewer Albrechtsen et al. (2016)
Semaglutide	Once weekly (subcutaneous injection) or daily oral administration	7 days	Good tolerance in the elderly, cardiovascular protection, stable blood glucose reduction	Nausea, constipation (low incidence)	Donnelly (2012), Razavi et al. (2022)
Dulaglutide	Once weekly (subcutaneous injection)	5.4 days	Most significant improvement in TIR, delays early nephropathy	Diarrhea, abdominal distension (transient)	Lymperopoulos et al. (2025), Romagnoli et al. (2001)
Polyethylene Glycol Loxenatide	Once weekly (subcutaneous injection)	5.5 days	No hypoglycemia when combined with metformin, weight-neutral	Injection site redness and swelling (3%–5%), mild nausea	Zou et al. (2025), Kumar (2025)
Benaglutide	Three times daily (subcutaneous injection before meals)	1.1 h	Controls postprandial blood glucose	Vomiting, abdominal pain (high incidence, requires initiation at a low dose)	Lymperopoulos et al. (2025), Romagnoli et al. (2001)

### 4.5 Adverse reactions of GLP-1RAs

The adverse reactions of GLP-1RAs are mainly gastrointestinal reactions (Liu et al., 2022), including common symptoms such as nausea, vomiting, diarrhea, abdominal distension, and decreased appetite. These reactions are mostly transient or alleviable by starting with a low dose, and the incidence of gastrointestinal reactions is relatively higher in short-acting formulations. For some injectable formulations, mild reactions such as injection site redness and swelling may occur (with an incidence of approximately 3%–5%) (Usach et al., 2019). In addition, since GLP-1RAs rely on promoting insulin secretion to exert their effects, they are not suitable for patients with T1DM or T2DM with complete pancreatic β-cell failure, and cannot replace insulin therapy (Wajchenberg, 2007).

## 5 Literature search and selection strategy

Literature retrieval covered public medicine database, PubMed, Embase, Web of Science and cochrane library, with core search terms including GLP-1RA and T2DM, supplemented by terms such as “molecular mechanism”, “clinical trial”, and “preclinical study”. Included were English full-text original studies and high-quality systematic reviews/meta-analyses focusing on the association between the two, excluded were non-T2DM models, conference abstracts without complete data, literature published before 2015, and duplicate publications (Sun et al., 2022). Two researchers independently screened the literature in the sequence of “title→abstract→full text”, with discrepancies resolved by a third researcher through arbitration, and the included studies were categorized and integrated by type (Van Harten, 2012).

## 6 Discussion

T2DM is a chronic metabolic disease with a high global incidence (Hameed et al., 2015), characterized by insulin resistance and progressive decline in pancreatic β-cell function as its core pathological features. The core advantage of GLP-1RAs lies in the establishment of a “multi-dimensional and low-risk” metabolic regulatory network. Its innovation is reflected not only in the enzymatic degradation resistance brought by structural modification, but also in the integration of a threefold mechanism, “classic pathways regulating glucose metabolism, cytokine signaling regulating energy metabolism, and DC buffer system regulating safety” — which perfectly aligns with the pathological nature of “metabolic syndrome” in T2DM. Long-term hyperglycemia is prone to inducing complications in multiple organs such as the heart, liver, and kidneys, significantly increasing the disability and mortality rates of patients (Kawahito et al., 2009). According to statistics from the International diabetes federation, in 2021, the number of T2DM patients aged 20–79 worldwide reached 537 million (Sun et al., 2022), and this number is expected to increase to 783 million by 2045, china accounts for nearly one-third of these patients, and only 49.2% of them can achieve the glycemic control target of HbA1c < 7.0% (Van Netten et al., 2024). Although traditional hypoglycemic drugs such as insulin and sulfonylureas can control blood glucose in the short term, they generally have limitations such as weight gain and increased risk of hypoglycemia, making it difficult to meet the clinical needs of “comprehensive metabolic management” for T2DM (Bailey et al., 2016). Against this background, GLP-1RA overcomes the metabolic shortcomings of endogenous GLP-1 through structural modification (Zheng et al., 2024). A “glucose concentration-dependent” multi-dimensional regulatory system was established, which not only achieves precise blood glucose lowering but also exerts effects such as weight loss and organ protection. This regulatory system has thus become a revolutionary breakthrough in the field of T2DM treatment. The core innovation of GLP-1RA lies in site-specific modification of the amino acid sequence (Zheng et al., 2025) (e.g., replacing alanine at position eight of natural GLP-1 with glycine or α-aminoisobutyric acid), which significantly enhances resistance to DPP-4 and extends the *in vivo* half-life to several hours to several days (Razavi et al., 2022), solving the problem that endogenous GLP-1 cannot be clinically applied due to rapid degradation (with a half-life of only 1–2 min) (Chun and Butts, 2020). From a molecular mechanism perspective, the regulation of glucose metabolism by GLP-1RA exhibits high specificity and synergy (Zhang Y. et al., 2025), in pancreatic β-cells, after binding to GLP-1R, it activates the Gs protein-coupled signaling pathway (Meloni et al., 2013), driving AC to catalyze the conversion of ATP to cAMP, the increased cAMP further activates PKA and EPAC. Among them, PKA promotes calcium ion influx by closing inward rectifier potassium channels (KIR6.2/SUR1) and opening VDCCs, thereby promoting the migration and membrane fusion of insulin-secreting vesicles (Yang et al., 2014), EPAC enhances the secretory effect by regulating the exocytosis of insulin granules (Seino et al., 2009). More importantly, this secretory-promoting process is strictly dependent on glucose concentration—when blood glucose exceeds the physiological threshold, the binding affinity of GLP-1RA to GLP-1R and the activation intensity of downstream signals are significantly enhanced, efficiently promoting insulin release (Moiz et al., 2025), when blood glucose returns to the normal range, the activation efficiency of the signaling pathway naturally decreases with the decline in blood glucose (Henquin, 2000), avoiding the risk of hypoglycemia caused by the “non-glucose-dependent” secretion promotion of traditional sulfonylurea drugs at the molecular level, which is also its core advantage distinguishing it from traditional secretagogues. In the regulation of pancreatic α-cells, GLP-1RA forms a dual network of “direct inhibition-indirect synergy”, at the direct level, after binding to GLP-1R on the α-cell membrane, it activates gi protein, inhibits AC activity, and reduces intracellular cAMP levels, as cAMP is a key second messenger for glucagon synthesis and secretion, the decrease in its level can directly inhibit the release of glucagon granules, at the indirect level, insulin secreted by β-cells under the promotion of GLP-1RA binds to insulin receptors on the α-cell membrane through paracrine action, activating the PI3K/Akt signaling pathway to further inhibit glucagon synthesis (Zhang Y. et al., 2025; Meir et al., 2025). This bidirectional regulatory mode enables GLP-1RA to not only lower blood glucose by promoting insulin secretion but also prevent excessive blood glucose fluctuations by inhibiting glucagon release (Essaouiba, 2020), perfectly matching the pathological core of “glucose metabolism imbalance” in T2DM. Beyond islet regulation, the improvement of insulin resistance by GLP-1RA exhibits multi-tissue targeting characteristics, covering three core metabolic organs, adipose tissue, liver, and skeletal muscle (Eizirik et al., 2020). In adipose tissue, GLP-1RA regulates the secretion balance of adipocytokines, promotes adiponectin release, and alleviates leptin resistance—adiponectin accelerates glucose uptake and oxidation in adipocytes by activating the AMPK pathway, while inhibiting lipolysis to reduce circulating FFA levels, thereby reducing the interference of free fatty acids with the IRS-1/PI3K/Akt insulin signaling pathway and enhancing insulin sensitivity, in liver tissue, GLP-1RA inhibits the activity of key gluconeogenic enzymes such as PEPCK and G6Pase, reduces hepatic glycogenolysis and gluconeogenesis, lowers endogenous glucose production, and simultaneously regulates hepatic lipid metabolism, reduces triglyceride accumulation in hepatocytes, and alleviates the damage of lipotoxicity to hepatic insulin sensitivity, in skeletal muscle tissue, GLP-1RA relies on the activation of the PI3K/Akt pathway to promote the upregulation of GLUT4 expression and its translocation to the cell membrane, accelerating the uptake and utilization of glucose by skeletal muscle cells and directly improving the responsiveness of skeletal muscle to insulin. This multi-organ synergistic regulatory mode allows GLP-1RA to improve the insulin resistance state of T2DM from the source, rather than simply relying on “exogenous supplementation” or “forced secretion” of insulin for symptomatic treatment. The progressive decline in pancreatic β-cell function is a core driving factor for T2DM progression (Zhang X. et al., 2025), and the “protective-reparative” dual effect of GLP-1RA on β-cells makes it go beyond the therapeutic scope of traditional hypoglycemic drugs. In terms of anti-apoptosis, GLP-1RA exerts its effects through three mechanisms (Xu et al., 2009), first, it downregulates the expression of pro-apoptotic proteins bad and bax and simultaneously upregulates the level of anti-apoptotic protein Bcl-2 by relying on the PI3K/Akt signaling pathway, maintaining the balance of apoptosis-related proteins, second, it enhances the activity of antioxidant enzymes such as SOD and CAT, reduces the production of ROS, and alleviates the damage of oxidative stress to β-cells, third, it inhibits the release of inflammatory mediators such as TNF-α and IL-6, reducing the damage of chronic low-grade inflammation to β-cells. In terms of promoting proliferation and differentiation, GLP-1RA can activate pathways such as PI3K/Akt and MAPK, promote the expression of proliferation-related genes including cyclin D1 (which promotes the transition of cells from G1 to S phase) and proliferating cell nuclear antigen (proliferating cell nuclear antigen (PCNA), involved in DNA replication), and simultaneously induce the directional differentiation of pancreatic endogenous stem cells and progenitor cells into functionally mature β-cells, enriching the source of β-cells. This dual effect of “quantity protection + function repair” on β-cells provides a molecular basis for delaying T2DM progression and even reversing early lesions, and is also a key feature distinguishing it from symptomatic treatment drugs such as insulin. From the perspective of clinical translation, the application of GLP-1RA has expanded from “poor glycemic control” to “comprehensive metabolic management”, and its clinical value is significantly reflected in combination therapy, special populations, and dosage form selection. In combination therapy, the combination of GLP-1RA and metformin exhibits the advantage of “mechanism complementarity”—metformin mainly improves insulin resistance in peripheral tissues, while GLP-1RA focuses on protecting islet function. From the perspective of mechanism synergy, the discovery of the IL-6/STAT3 pathway and the DC buffer system has filled the gaps in traditional research, the IL-6/STAT3 pathway extends the regulatory dimension of GLP-1RAs from “simple glucose lowering” to “enhanced energy expenditure” (Alharbi, 2024). It achieves the synergy between weight loss and glucose control through adipose tissue browning, while the DC buffer system, as revealed in relevant literature can regulate the persistence and action range of IL-6, enabling the GLP-1RA-induced IL-6 to only target adipose tissue for exerting effects without triggering systemic inflammation (Vallon and Thomson, 2017) which is also a key feature distinguishing GLP-1RAs from other IL-6-promoting drugs, their combined application can increase the HbA1c reduction by 0.5%–1.0% without the risk of weight gain, the combination with sodium-glucose cotransporter 2 (SGLT2) inhibitors (e.g., dapagliflozin) can superimpose cardiovascular and renal protective effects, GLP-1RA improves vascular endothelial function through anti-inflammation and antioxidant effects, while SGLT2 inhibitors reduce intraglomerular pressure by reducing glucose reabsorption (Huynh et al., 2023), and the two synergistically reduce the risk of cardiovascular events and renal failure in T2DM patients, aligning with the current therapeutic goal of T2DM focusing on “complication prevention and control”. In special populations, the individualized advantages of GLP-1RA are particularly prominent, elderly T2DM patients have good tolerance to semaglutide (Gasmi et al., 2023), which not only effectively lowers blood glucose but also reduces vascular endothelial damage by inhibiting oxidative stress and inflammatory responses, thereby reducing the risk of cardiovascular complications in elderly patients, overweight/obese T2DM patients show a more significant response to benaglutide (Moiz et al., 2025), which inhibits the central appetite regulation center and delays gastric emptying rate, achieving weight loss while controlling postprandial blood glucose with an average weight loss of 3–5 kg, effectively improving obesity-related insulin resistance, for patients with significant postprandial blood glucose fluctuations, short-acting GLP-1RA (e.g., benaglutide) is more likely to match the changes in postprandial blood glucose peaks due to its rapid onset and short duration of action (Zhou et al., 2025), long-acting formulations (e.g., dulaglutide, semaglutide) significantly improve patient medication adherence due to their once-weekly administration frequency, and have more stable blood drug concentrations, longer-lasting hypoglycemic effects, and the most significant improvement in TIR, among them, dulaglutide has also been proven to delay the progression of early diabetic nephropathy, further expanding its organ protection value. However, GLP-1RA still faces challenges to be addressed in clinical application and mechanism research. At the mechanism level, some pathways of its organ protection effect have not been fully clarified, for example, in the process of liver protection, the specific molecular association between the downregulation of miR-34a/21 and upregulation of miR-200b/c by GLP-1RA and the improvement of hepatic lipid metabolism has only been verified in animal models, lacking further confirmation from clinical samples, in the process of renal protection, the cross-linking mechanism between the PKA/PKG signaling pathway and the improvement of renal artery endothelial function has not yet been fully elucidated, thus, more *in vivo* experiments are still needed to explore the interaction between the signaling molecules involved in this process. In addition, drugs such as liraglutide exhibit “effect attenuation” in central appetite inhibition during long-term application (e.g., weakened inhibitory effect after 12 weeks of treatment), which suggests the possible existence of GLP-1R desensitization or body compensation mechanisms. However, the molecular basis of this “effect attenuation” (e.g., downregulation of GLP-1R expression) remains to be further clarified. Changes in the activity of downstream signal adaptor proteins) and response strategies (e.g., dose adjustment, combination medication) still need in-depth research. At the clinical practice level, gastrointestinal reactions are the main factor limiting the application of GLP-1RA, although the administration method of starting with a low dose and gradually increasing the dose can alleviate some discomfort, 5%–10% of patients still discontinue treatment due to adverse reactions such as nausea, vomiting, and diarrhea, how to reduce gastrointestinal irritation through dosage form improvement or combination medication is the key to improving patient tolerance. At the same time, GLP-1RA has obvious therapeutic limitations, as it relies on promoting insulin secretion to exert its effect, it is not applicable to patients with type 1 diabetes or T2DM patients with complete failure of pancreatic β-cell function, and cannot replace the role of insulin in the diabetes treatment system, existing cost-effectiveness data are mostly based on short-term (6–12 months) clinical studies, and the long-term (more than 5 years) economic impact of long-acting GLP-1RA on complications such as diabetic nephropathy and cardiovascular events still needs further verification through large-sample, multi-center cohort studies, especially in regions with limited medical resources, there is still controversy over whether its cost-effectiveness ratio is better than that of traditional drugs. To address the above challenges, future research on GLP-1RA can seek breakthroughs in three aspects, first, dosage form improvement—on the basis of the launch of oral formulations (e.g., oral semaglutide), further optimize delivery systems (e.g., lipid nanoparticles, enteric coating technology) to improve oral bioavailability, and develop targeted delivery systems (e.g., liver-targeted, pancreas-targeted nanoparticles) to reduce the distribution of drugs in non-target organs and lower the incidence of adverse reactions such as gastrointestinal reactions, second, mechanism deepening—explore the interaction between GLP-1RA and intestinal microecology (e.g., the regulatory effect of short-chain fatty acids on GLP-1 secretion, the impact of changes in flora composition on the efficacy of GLP-1RA) and the cross-talk with longevity-related signaling pathways such as the Sirtuin family, providing a theoretical basis for expanding its indications in metabolic-related diseases such as NASH and alzheimer’s disease. In precision medicine practice, a prediction model is established based on the genotyping of diabetes susceptibility genes such as combined with patient metabolic indicators (e.g., insulin resistance index HOMA-IR, β-cell function index HOMA-β), aiming to screen patient groups with better response to GLP-1RAs. This approach is designed to realize “individualized medication” in the clinical application of GLP-1RAs for T2DM treatment. avoid unnecessary treatment waste, and at the same time accumulate medication experience in patients with different comorbidities (e.g., hypertension, chronic kidney disease) through real-world data registries to guide drug selection in complex clinical scenarios. In summary, GLP-1RA overcomes the metabolic shortcomings of endogenous GLP-1 through structural modification[119], and with a “glucose concentration-dependent” precise regulatory mode, achieves multi-dimensional intervention in the pathological links of T2DM (insulin resistance, β-cell decline, glucose metabolism imbalance), while having effects such as weight loss and organ protection, completely reshaping the treatment pattern of T2DM. However, in the process from “basic research” to “clinical translation”, it is still necessary to overcome challenges such as unknown mechanisms, insufficient tolerance, and unclear cost-effectiveness. In the future, through closed-loop research of “mechanism analysis-clinical verification-translational application”, the efficacy and safety of GLP-1RA will be further optimized, which is expected to enable it to play a more core role in the comprehensive management of T2DM and related metabolic diseases (e.g., obesity, NASH), providing a more complete treatment strategy for global diabetes prevention and control.

## 7 Conclusion

With the widespread application of GLP-1RA in T2DM treatment, the advantages of its multi-targeted and multi-organ synergistic effects have become increasingly prominent. Through structural modification, GLP-1RA effectively extends its half-life, overcomes the defect that endogenous GLP-1 is easily degraded by DPP-4, and establishes a glucose concentration-dependent precise regulatory network, achieving multiple effects including blood glucose lowering, weight loss, β-cell protection, and multi-organ function preservation. GLP-1RAs establish a regulatory network consisting of “classic pathways (cyclic AMP-PKA/phosphoinositide 3-kinase-Akt/AMP-activated protein kinase, cAMP-PKA/PI3K-Akt/AMPK)+IL-6/STAT3 cytokine signaling + DC blood buffer system”, thereby achieving comprehensive treatment for T2DM with effects of glucose lowering, organ protection, and weight loss. In clinical practice, their monotherapy or combination therapy (e.g., with metformin) can significantly reduce HbA1c, and they are particularly suitable for elderly and obese patients. In the future, it is necessary to further deepen the analysis of the DC buffer mechanism, develop individualized regimens based on biomarkers, and promote their transition from “glucose-lowering drugs” to “metabolic regulation platforms”. Clinical studies have shown that GLP-1RA, either as monotherapy or in combination with sodium-glucose cotransporter two inhibitors, metformin, and other drugs, can significantly reduce HbA1c, lower the risk of hypoglycemia, and exhibit more significant efficacy in elderly patients, obese patients, and those with large blood glucose fluctuations. Meanwhile, GLP-1RA has also shown potential protective effects in the liver, kidneys, and cardiovascular system, providing a theoretical basis for its expanded application in metabolic syndrome and its complications. Although current application data in special populations is still limited, and some mechanisms—such as miRNA-regulated lipid metabolism and PKA/PKG-mediated renal protective pathways—still require further clinical verification, GLP-1RA, as an important tool for precise T2DM treatment, will continue to strengthen its core position in diabetes and related metabolic diseases through structural optimization, dosage form improvement, and the development of multi-target agonists. In the future, research on its individualized treatment, combined medication strategies, and long-term safety should be strengthened to promote the transformation of GLP-1RA from a “hypoglycemic drug” to a “metabolic integration and regulation platform,” providing more comprehensive, sustainable, and safe treatment options for T2DM patients.

## References

[B1] Aaboe K. Krarup T. Madsbad S. Holst J. J. (2008). GLP‐1, physiological effects and potential therapeutic applications. Diabetes, Obesity Metabolism 10 (11), 994–1003. 10.1111/j.1463-1326.2008.00853.x 18435775

[B2] Alharbi S. H. (2024). Anti-inflammatory role of glucagon-like peptide 1 receptor agonists and its clinical implications. Ther. Advances Endocrinology Metabolism 15, 20420188231222367. 10.1177/20420188231222367 38288136 PMC10823863

[B3] Anyiam O. Phillips B. Quinn K. Wilkinson D. Smith K. Atherton P. (2024). Metabolic effects of very-low calorie diet, semaglutide, or combination of the two, in individuals with type 2 diabetes mellitus. Clin. Nutr. 43 (8), 1907–1913. 10.1016/j.clnu.2024.06.034 38996661

[B4] Aperia A. Holtbäck U. Syrén M. L. Svensson L. B. Fryckstedt J. Greengard P. (1994). Activation/deactivation of renal Na+, K+‐ATPase, a final common pathway for regulation of natriuresis. FASEB Journal 8 (6), 436–439. 10.1096/fasebj.8.6.8168694 8168694

[B5] Ashik T. (2023). Identifying alterations in adipose tissue-derived islet GPCR peptide ligand mRNAs in obesity, implications for islet function. London: Ph. D. thesis. King’s College.

[B6] Author anonymous Type 2 diabetes mellitus (T2DM) accounts for more than 90% of all diabetes cases, while its mechanism is centered on insulin resistance and progressive pancreatic beta-cell dysfunction (2017).

[B7] Baggio L. L. Drucker D. J. (2007). Biology of incretins, GLP-1 and GIP. Gastroenterology 132 (6), 2131–2157. 10.1053/j.gastro.2007.03.054 17498508

[B8] Bailey C. J. Tahrani A. A. Barnett A. H. (2016). Future glucose-lowering drugs for type 2 diabetes. Lancet Diabetes and Endocrinology 4 (4), 350–359. 10.1016/S2213-8587(15)00462-3 26809680

[B9] Bargut T. C. L. Souza-Mello V. Aguila M. B. Mandarim-de-Lacerda C. A. (2017). Browning of white adipose tissue: lessons from experimental models. Hormone Molecular Biology Clinical Investigation 31 (1), 20160051. 10.1515/hmbci-2016-0051 28099124

[B10] Brubaker P. L. Drucker D. J. (2002). Structure-function of the glucagon receptor family of G protein-coupled receptors, the glucagon, GIP, GLP-1, and GLP-2 receptors. Recept. Channels 8 (3-4), 179–188. 10.1080/10606820213687 12529935

[B11] Camaya I. Donnelly S. O'Brien B. (2022). Targeting the PI3K/Akt signaling pathway in pancreatic β‐cells to enhance their survival and function, an emerging therapeutic strategy for type 1 diabetes. J. Diabetes 14 (4), 247–260. 10.1111/1753-0407.13252 35191175 PMC9060113

[B12] Changeux J. P. Christopoulos A. (2016). Allosteric modulation as a unifying mechanism for receptor function and regulation. Cell 166 (5), 1084–1102. 10.1111/dom.12959 27565340

[B13] Chaudhury A. Duvoor C. Reddy Dendi V. S. Kraleti S. Chada A. Ravilla R. (2017). Clinical review of antidiabetic drugs, implications for type 2 diabetes mellitus management. Front. Endocrinology 8, 6. 10.3389/fendo.2017.00006 28167928 PMC5256065

[B14] Chun J. H. Butts A. (2020). Long-acting GLP-1 receptor agonists: an overview of efficacy, safety, and their role in type 2 diabetes management. JAAPA 33 (S8), 3–18. 10.1097/01.JAA.0000669456.13763.bd 32756220

[B15] Donnelly D. (2012). The structure and function of the glucagon‐like peptide‐1 receptor and its ligands. Br. Journal Pharmacology 166 (1), 27–41. 10.1111/j.1476-5381.2011.01687.x 21950636 PMC3415635

[B16] Drucker D. J. (2022). GLP-1 physiology informs the pharmacotherapy of obesity. Mol. Metabolism 57, 101351. 10.1016/j.molmet.2021.101351 34626851 PMC8859548

[B17] Eguchi N. Vaziri N. D. Dafoe D. C. Ichii H. (2021). The role of oxidative stress in pancreatic β cell dysfunction in diabetes. Int. Journal Molecular Sciences 22 (4), 1509. 10.3390/ijms22041509 33546200 PMC7913369

[B18] Einarson T. R. Acs A. Ludwig C. Panton U. H. (2018). Prevalence of cardiovascular disease in type 2 diabetes, a systematic literature review of scientific evidence from across the world in 2007–2017. Cardiovasc. Diabetology 17 (1), 83. 10.1186/s12933-018-0728-6 29884191 PMC5994068

[B19] Eizirik D. L. Pasquali L. Cnop M. (2020). Pancreatic β-cells in type 1 and type 2 diabetes mellitus, different pathways to failure. Nat. Reviews Endocrinology 16 (7), 349–362. 10.1038/s41574-020-0355-7 32398822

[B20] Essaouiba A. (2020). Development of a liver-pancreas *in vitro* model using microfluidic organ-on-chip technologies[D]. Univ. Technol. compiègne. Available online at: https://theses.hal.science/tel-03234302v1

[B21] Farré R. Tack J. (2013). Food and symptom generation in functional gastrointestinal disorders, physiological aspects. Official Journal American College Gastroenterology| ACG 108 (5), 698–706. 10.1038/ajg.2013.24 23458851

[B22] Galicia-Garcia U. Benito-Vicente A. Jebari S. Larrea-Sebal A. Siddiqi H. Uribe K. B. (2020). Pathophysiology of type 2 diabetes mellitus. Int. Journal Molecular Sciences 21 (17), 6275. 10.3390/ijms21176275 32872570 PMC7503727

[B23] Gasmi A. Mujawdiya P. K. Nehaoua A. Shanaida M. Semenova Y. Piscopo S. (2023). Pharmacological treatments and natural biocompounds in weight management. Pharm. (Basel) 16 (2), 212. 10.3390/ph16020212 37139804 PMC9962258

[B24] Ghosh P. Fontanella R. A. Scisciola L. Pesapane A. Taktaz F. Franzese M. (2023). Targeting redox imbalance in neurodegeneration, characterizing the role of GLP-1 receptor agonists. Theranostics 13 (14), 4872–4884. 10.7150/thno.86831 37771773 PMC10526673

[B25] Gourdy P. Darmon P. Dievart F. Halimi J. M. Guerci B. (2023). Combining glucagon-like peptide-1 receptor agonists (GLP-1RAs) and sodium-glucose cotransporter-2 inhibitors (SGLT2is) in patients with type 2 diabetes mellitus (T2DM). Cardiovasc. Diabetology 22 (1), 79. 10.1186/s12933-023-01798-4 37005640 PMC10067319

[B26] Grieco M. Giorgi A. Gentile M. C. d'Erme M. Morano S. Maras B. (2019). Glucagon-like peptide-1, a focus on neurodegenerative diseases. Front. Neuroscience 13, 1112. 10.3389/fnins.2019.01112 31680842 PMC6813233

[B27] Habib S. H. Saha S. (2010). Burden of non-communicable disease, global overview. Diabetes and Metabolic Syndrome, Clin. Res. and Rev. 4 (1), 41–47. 10.1016/j.dsx.2008.04.005

[B28] Hameed I. Masoodi S. R. Mir S. A. Nabi M. Ghazanfar K. Ganai B. A. (2015). Type 2 diabetes mellitus, from a metabolic disorder to an inflammatory condition. World Journal Diabetes 6 (4), 598–612. 10.4239/wjd.v6.i4.598 25987957 PMC4434080

[B29] He X. Zhao W. Li P. H. Zhang Y. Li G. Su H. (2025). Research progress of GLP-1RAs in the treatment of type 2 diabetes mellitus. Front. Pharmacology 15, 1483792. 10.3389/fphar.2024.1483792 39902077 PMC11788294

[B30] Hedeskov C. J. (1980). Mechanism of glucose-induced insulin secretion. Physiol. Reviews 60 (2), 442–509. 10.1152/physrev.1980.60.2.442 6247727

[B31] Henquin J. C. (2000). Triggering and amplifying pathways of regulation of insulin secretion by glucose. Diabetes 49 (11), 1751–1760. 10.2337/diabetes.49.11.1751 11078440

[B32] Hohenester E. Engel J. (2002). Domain structure and organisation in extracellular matrix proteins. Matrix Biol. 21 (2), 115–128. 10.1016/s0945-053x(01)00191-3 11852228

[B33] Hou J. C. Min L. Pessin J. E. (2009). Insulin granule biogenesis, trafficking and exocytosis. Vitam. Horm. 80, 473–506. 10.1016/S0083-6729(08)00616-X 19251047 PMC4324607

[B34] Huynh G. Runeberg H. Weideman R. (2023). Evaluating weight loss with semaglutide in elderly patients with type II diabetes. J. Pharm. Technol. 39 (1), 10–15. 10.1177/87551225221137493 36755752 PMC9899961

[B35] Idris I. (2019). News and views July 2019. Diabetes. Obes. and Metabolism 21 (7). 10.1111/dom.13772 31144446

[B36] Ilonen J. Lempainen J. Veijola R. (2019). The heterogeneous pathogenesis of type 1 diabetes mellitus. Nat. Reviews Endocrinology 15 (11), 635–650. 10.1038/s41574-019-0254-y 31534209

[B37] Jalleh R. J. Rayner C. K. Hausken T. Jones K. L. Camilleri M. Horowitz M. (2024). Gastrointestinal effects of GLP-1 receptor agonists, mechanisms, management, and future directions. Lancet Gastroenterology and Hepatology 9 (10), 957–964. 10.1016/S2468-1253(24)00188-2 39096914

[B38] James W. P. (2008). WHO recognition of the global obesity epidemic. Int. J. Obes. (Lond). 32 (Suppl. 7), S120–S126. 10.1038/ijo.2008.247 19136980

[B39] Jones B. Buenaventura T. Kanda N. Chabosseau P. Owen B. M. Scott R. (2018). Targeting GLP-1 receptor trafficking to improve agonist efficacy. Nat. Communications 9 (1), 1602. 10.1038/s41467-018-03941-2 29686402 PMC5913239

[B40] Katsarou A. Gudbjörnsdottir S. Rawshani A. Dabelea D. Bonifacio E. Anderson B. J. (2017). Type 1 diabetes mellitus. Nat. Reviews Disease Primers 3 (1), 1–17. 10.1038/nrdp.2017.16 28358037

[B41] Kawahito S. Kitahata H. Oshita S. (2009). Problems associated with glucose toxicity, role of hyperglycemia-induced oxidative stress. World Journal Gastroenterology, WJG 15 (33), 4137–4142. 10.3748/wjg.15.4137 19725147 PMC2738809

[B42] Kumar V. (2025). GLP-1/GLP-1R axis: from metabolism (obesity and T2DM) to immunity. Open Biol. 15 (7), 240303. 10.1098/rsob.240303 40592472 PMC12212992

[B43] Lam S. M. Wang Z. Song J. W. Shi Y. Liu W. Y. Wan L. Y. (2025). Non-invasive lipid panel of MASLD fibrosis transition underscores the role of lipoprotein sulfatides in hepatic immunomodulation. Cell Metab. 37 (1), 69–86. e7. 10.1016/j.cmet.2024.09.009 39500328

[B44] Lang J. (1999). Molecular mechanisms and regulation of insulin exocytosis as a paradigm of endocrine secretion. Eur. Journal Biochemistry 259 (1‐2), 3–17. 10.1046/j.1432-1327.1999.00043.x 9914469

[B45] Leite Â. (2025). Chronic illnesses, varied health patterns and mental health challenges[C]. Healthcare 13 (12), 1396. 10.3390/healthcare13121396 40565424 PMC12192847

[B46] Lim G. E. Brubaker P. L. (2006). Glucagon-like peptide 1 secretion by the L-cell, the view from within. Diabetes 55 (Suppl. ment_2), S70–S77. 10.2337/db06-s020

[B47] Liu L. Chen J. Wang L. Chen C. (2022). Association between different GLP-1 receptor agonists and gastrointestinal adverse reactions: a real-world disproportionality study based on FDA adverse event reporting system database. Front. Endocrinology 13, 1043789. 10.3389/fendo.2022.1043789 36568085 PMC9770009

[B48] Lotfy M. Adeghate J. Kalasz H. Singh J. Adeghate E. (2017). Chronic complications of diabetes mellitus, a mini review. Curr. Diabetes Reviews 13 (1), 3–10. 10.2174/1573399812666151016101622 26472574

[B49] Lymperopoulos A. Altsman V. L. Stoicovy R. A. (2025). Glucagon-like peptide-1 receptor (GLP-1R) signaling, making the case for a functionally gs protein-selective GPCR. Int. Journal Molecular Sciences 26 (15), 7239. 10.3390/ijms26157239 40806371 PMC12347461

[B50] Ma X. Liu Z. Ilyas I. Little P. J. Kamato D. Sahebka A. (2021). GLP-1 receptor agonists (GLP-1RAs), cardiovascular actions and therapeutic potential. Int. Journal Biological Sciences 17 (8), 2050–2068. 10.7150/ijbs.59965 34131405 PMC8193264

[B51] Mei F. C. Qiao J. Tsygankova O. M. Meinkoth J. L. Quilliam L. A. Cheng X. (2002). Differential signaling of cyclic AMP, opposing effects of exchange protein directly activated by cyclic AMP and cAMP-dependent protein kinase on protein kinase B activation. J. Biological Chemistry 277 (13), 11497–11504. 10.1074/jbc.M110856200 11801596

[B52] Meir K. Niznik S. Avnery O. Zoref-Lorenz A. Agmon-Levin N. Ellis M. H. (2025). Vitamin K antagonist anticoagulation in antiphospholipid syndrome, time in therapeutic range and clinical outcomes. Am. Journal Medicine 138 (2), 269–276. e1. 10.1016/j.amjmed.2024.09.019 39362574

[B53] Meloni A. R. DeYoung M. B. Lowe C. Parkes D. G. (2013). GLP-1 receptor activated insulin secretion from pancreatic β-cells, mechanism and glucose dependence. Diabetes Obes. Metab. 15 (1), 15–27. 10.1111/j.1463-1326.2012.01663.x 22776039 PMC3556522

[B54] Moiz A. Filion K. B. Tsoukas M. A. Yu O. H. Peters T. M. Eisenberg M. J. (2025). Mechanisms of GLP-1 receptor agonist-induced weight loss: A review of central and peripheral pathways in appetite and energy regulation. Am. Journal Medicine 138 (6), 934–940. 10.1016/j.amjmed.2025.01.021 39892489

[B55] Monti G. Gomes Moreira D. Richner M. Mutsaers H. A. M. Ferreira N. Jan A. (2022). GLP-1 receptor agonists in neurodegeneration, neurovascular unit in the spotlight. Cells 11 (13), 2023. 10.3390/cells11132023 35805109 PMC9265397

[B56] Mu W. Cheng X. F. Liu Y. Lv Q. Z. Liu G. L. Zhang J. G. (2019). Potential nexus of non-alcoholic fatty liver disease and type 2 diabetes mellitus: insulin resistance between hepatic and peripheral tissues. Front. Pharmacology 9, 1566. 10.3389/fphar.2018.01566 30692925 PMC6339917

[B57] Mulvihill E. E. Drucker D. J. (2014). Pharmacology, physiology, and mechanisms of action of dipeptidyl peptidase-4 inhibitors. Endocr. Reviews 35 (6), 992–1019. 10.1210/er.2014-1035 25216328 PMC7108477

[B58] Nauck M. A. Quast D. R. Wefers J. Meier J. J. (2022). GLP-1 receptor agonists in the treatment of type 2 diabetes – status 2022. Cardiovasc Diabetol. 21, 159. 10.1186/s12933-022-01604-7 35996111

[B59] Nevola R. Epifani R. Imbriani S. Tortorella G. Aprea C. Galiero R. (2023). GLP-1 receptor agonists in non-alcoholic fatty liver disease, current evidence and future perspectives. Int. J. Mol. Sci. 24 (2), 1703. 10.3390/ijms24021703 36675217 PMC9865319

[B60] Notaro N. (2024). Unacylated ghrelin stimulates fatty acid oxidation in isolated skeletal muscle from rats receiving high-intensity exercise, regardless of dietary fat intake[D]. Guelph, Canada: University of guelph.

[B61] Owen M. R. Doran E. Halestrap A. P. (2000). Evidence that metformin exerts its anti-diabetic effects through inhibition of complex 1 of the mitochondrial respiratory chain. Biochem. J. 347 (3), 607–614. 10.1042/bj3480607 10839993 PMC1221104

[B62] Pabreja K. Mohd M. A. Koole C. Wootten D. Furness S. G. B. (2014). Molecular mechanisms underlying physiological and receptor pleiotropic effects mediated by GLP‐1R activation. Br. Journal Pharmacology 171 (5), 1114–1128. 10.1111/bph.12313 23889512 PMC3952792

[B63] Petrovic A. Igrec D. Rozac K. Bojanic K. Kuna L. Kolaric T. O. (2023). The role of GLP1-RAs in direct modulation of lipid metabolism in hepatic tissue as determined using *in vitro* models of NAFLD. Curr. Issues Molecular Biology 45 (6), 4544–4556. 10.3390/cimb45060288 37367037 PMC10296833

[B64] Qaseem A. Wilt T. J. Kansagara D. Horwitch C. Barry M. J. Forciea M. A. (2018). Hemoglobin A1c targets for glycemic control with pharmacologic therapy for nonpregnant adults with type 2 diabetes mellitus, a guidance statement update from the American college of physicians. Ann. Internal Medicine 168 (8), 569–576. 10.7326/M17-0939 29507945

[B65] Quesada I. Tudurí E. Ripoll C. Nadal A. (2008). Physiology of the pancreatic α-cell and glucagon secretion, role in glucose homeostasis and diabetes. J. Endocrinology 199 (1), 5–19. 10.1677/JOE-08-0290 18669612

[B66] Ramracheya R. Ward C. Shigeto M. Walker J. N. Amisten S. Zhang Q. (2010). Membrane potential-dependent inactivation of voltage-gated ion channels in α-cells inhibits glucagon secretion from human islets. Diabetes 59 (9), 2198–2208. 10.2337/db09-1505 20547976 PMC2927942

[B67] Razavi M. Wei Y. Y. Rao X. Q. Zhong J. X. (2022). DPP-4 inhibitors and GLP-1RAs, cardiovascular safety and benefits. Mil. Med. Res. 9 (1), 45. 10.1186/s40779-022-00410-2 35986429 PMC9392232

[B68] Ren X. Hua H. Wu Y. Zhang W. Long X. Bai Y. (2025). Efficacy and safety of GLP-1 agonists in the treatment of T2DM, A systematic review and network meta-analysis. Sci. Rep. 15 (1), 1–15. 10.1038/s41598-025-09807-0 40619508 PMC12230154

[B69] Romagnoli C. Frezza S. Cingolani A. De Luca A. Puopolo M. De Carolis M. P. (2001). Plasma levels of interleukin-6 and interleukin-10 in preterm neonates evaluated for sepsis. Eur. Journal Pediatrics 160 (6), 345–350. 10.1007/pl00008445 11421413

[B70] Sandoval D. A. D'Alessio D. A. (2015). Physiology of proglucagon peptides, role of glucagon and GLP-1 in health and disease. Physiol. Reviews 95 (2), 513–548. 10.1152/physrev.00013.2014 25834231

[B71] Saraiva F. K. Sposito A. C. (2014). Cardiovascular effects of glucagon-like peptide 1 (GLP-1) receptor agonists. Cardiovasc. Diabetology 13 (1), 142. 10.1186/s12933-014-0142-7 25338737 PMC4216654

[B72] Schueller F. Roy S. Vucur M. Trautwein C. Luedde T. Roderburg C. (2018). The role of miRNAs in the pathophysiology of liver diseases and toxicity. Int. Journal Molecular Sciences 19 (1), 261. 10.3390/ijms19010261 29337905 PMC5796207

[B73] Seino S. Takahashi H. Fujimoto W. Shibasaki T. (2009). Roles of cAMP signalling in insulin granule exocytosis. Diabetes, Obesity Metabolism 11, 180–188. 10.1111/j.1463-1326.2009.01108.x 19817800

[B74] Sesti G. (2006). Pathophysiology of insulin resistance. Best Practice and Research Clin. Endocrinology and Metabolism 20 (4), 665–679. 10.1016/j.beem.2006.09.007 17161338

[B75] Shilleh A. H. Viloria K. Broichhagen J. Campbell J. E. Hodson D. J. (2024). GLP1R and GIPR expression and signaling in pancreatic alpha cells, beta cells and delta cells. Peptides 175, 171179. 10.1016/j.peptides.2024.171179 38360354 PMC7618508

[B76] Sun H. Saeedi P. Karuranga S. Pinkepank M. Ogurtsova K. Duncan B. B. (2022). IDF diabetes atlas: global, regional and country-level diabetes prevalence estimates for 2021 and projections for 2045. Diabetes Research Clinical Practice 183, 109119. 10.1016/j.diabres.2021.109119 34879977 PMC11057359

[B77] Tengholm A. Gylfe E. cAMP signalling in insulin and glucagon secretion. Diabetes Obes. Metabolism (2017). 19, 42–53. 10.1111/dom.12993 28466587

[B78] Tomlinson B. Patil N. G. Fok M. Chan P. Lam C. W. K. (2022). The role of sulfonylureas in the treatment of type 2 diabetes. Expert Opinion Pharmacotherapy 23 (3), 387–403. 10.1080/14656566.2021.1999413 34758676

[B79] Tong J. C. L. Frazer-Morris C. Shilleh A. H. Viloria K. de Bray A. Nair A. M. (2025). Localized GLP1 receptor pre-internalization directs pancreatic alpha cell to beta cell communication. Cell Metab. 37 (8), 1698–1714. e5. 10.1016/j.cmet.2025.06.009 40664215 PMC12276844

[B80] Toth P. P. Fazio S. Wong N. D. Hull M. Nichols G. A. (2020). Risk of cardiovascular events in patients with hypertriglyceridaemia, A review of real‐world evidence. Diabetes, Obesity Metabolism 22 (3), 279–289. 10.1111/dom.13921 31742844 PMC7065050

[B81] Turnock L. A. Hearne E. Lazuras L. (2025). Made in China, the international supply of illicit semaglutide and weight-loss medicines online. Emerg. Trends Drugs, Addictions, Health 5, 100169. 10.1016/j.etdah.2024.100169

[B82] Usach I. Martinez R. Festini T. Peris J. E. (2019). Subcutaneous injection of drugs: literature review of factors influencing pain sensation at the injection site. Adv. Therapy 36 (11), 2986–2996. 10.1007/s12325-019-01101-6 31587143 PMC6822791

[B83] Vallon V. Thomson S. C. (2017). Targeting renal glucose reabsorption to treat hyperglycaemia, the pleiotropic effects of SGLT2 inhibition. Diabetologia 60 (2), 215–225. 10.1007/s00125-016-4157-3 27878313 PMC5884445

[B84] Van Harten G. (2012). Arbitrator behaviour in asymmetrical adjudication: an empirical study of investment treaty arbitration. Osgoode Hall. LJ 50, 211–268. 10.60082/2817-5069.1036

[B85] Van Netten J. J. Bus S. A. Apelqvist J. Chen P. Chuter V. Fitridge R. (2024). Definitions and criteria for diabetes‐related foot disease (IWGDF 2023 update). Diabetes/metabolism Research Reviews 40 (3), e3654. 10.1002/dmrr.3654 37186781

[B86] Vergès B. Aboyans V. Angoulvant D. Boutouyrie P. Cariou B. Hyafil F. (2022). Protection against stroke with glucagon-like peptide-1 receptor agonists, a comprehensive review of potential mechanisms. Cardiovasc. Diabetology 21 (1), 242. 10.1186/s12933-022-01686-3 36380358 PMC9667639

[B87] Wajchenberg B. L. (2007). β-cell failure in diabetes and preservation by clinical treatment. Endocr. Reviews 28 (2), 187–218. 10.1210/10.1210/er.2006-0038 17353295

[B88] Wang J. Wang H. (2017). Oxidative stress in pancreatic beta cell regeneration. Oxidative Medicine Cellular Longevity 2017 (1), 1930261. 10.1155/2017/1930261 28845211 PMC5560096

[B89] Wang J. Y. Wang Q. W. Yang X. Y. Yang W. Li D. R. Jin J. Y. (2023). GLP− 1 receptor agonists for the treatment of obesity: role as a promising approach. Front. Endocrinology 14, 1085799. 10.3389/fendo.2023.1085799 36843578 PMC9945324

[B90] Warwar N. Efendic S. Ostenson C. G. Haber E. P. Cerasi E. Nesher R. (2006). Dynamics of glucose-induced localization of PKC isoenzymes in pancreatic β-cells, diabetes-related changes in the GK rat. Diabetes 55 (3), 590–599. 10.2337/diabetes.55.03.06.db05-0001 16505220

[B91] Wen S. Nguyen T. Gong M. Yuan X. Wang C. Jin J. (2021). An overview of similarities and differences in metabolic actions and effects of central nervous system between glucagon-like peptide-1 receptor agonists (GLP-1RAs) and sodium glucose co-transporter-2 inhibitors (SGLT-2is). Diabetes, Metabolic Syndrome Obesity 14, 2955–2972. 10.2147/DMSO.S312527 34234493 PMC8254548

[B92] Wewer Albrechtsen N. J. Kuhre R. E. Pedersen J. Knop F. K. Holst J. J. (2016). The biology of glucagon and the consequences of hyperglucagonemia. Biomarkers Medicine 10 (11), 1141–1151. 10.2217/bmm-2016-0090 27611762

[B93] Wu Y. Wang Z. Zhao Z. Song X. Miao M. Zhang X. (2023). Bile acid metabolites in early pregnancy and risk of gestational diabetes mellitus, results from a prospective cohort study. Diabetes, Obesity Metabolism 25 (8), 2255–2267. 10.1111/dom.15104 37161712

[B94] Xu J. Lloyd D. J. Hale C. Stanislaus S. Chen M. Sivits G. (2009). Fibroblast growth factor 21 reverses hepatic steatosis, increases energy expenditure, and improves insulin sensitivity in diet-induced Obese mice. Diabetes 58 (1), 250–259. 10.2337/db08-0392 18840786 PMC2606881

[B95] Yang S. N. Shi Y. Yang G. Li Y. Yu J. Berggren P. O. (2014). Ionic mechanisms in pancreatic β cell signaling. Cell. Molecular Life Sciences 71 (21), 4149–4177. 10.1007/s00018-014-1680-6 25052376 PMC11113777

[B96] Yao H. Zhang A. Li D. Wu Y. Wang C. Z. Wan J. Y. (2024). Comparative effectiveness of GLP-1 receptor agonists on glycaemic control, body weight, and lipid profile for type 2 diabetes: systematic review and network meta-analysis. BMJ, 384, 1–18. 10.1136/bmj-2023-076410 38286487 PMC10823535

[B97] Yousif A. S. Ronsard L. Shah P. Omatsu T. Sangesland M. Bracamonte Moreno T. (2021). The persistence of interleukin-6 is regulated by a blood buffer system derived from dendritic cells. Immunity 54 (2), 235–246. e5. 10.1016/j.immuni.2020.12.001 33357409 PMC7836640

[B98] Zaccardi F. Webb D. R. Yates T. Davies M. J. (2016). Pathophysiology of type 1 and type 2 diabetes mellitus, a 90-year perspective. Postgrad. Medical Journal 92 (1084), 63–69. 10.1136/postgradmedj-2015-133281 26621825

[B99] Zhang Z. Du Q. Jia H. Li Y. M. Liu Y. Q. Li S. B. (2024). A qualitative study on inner experience of self-management behavior among elderly patients with type 2 diabetes in rural areas. BMC Public Health 24 (1), 1456. 10.1186/s12889-024-18994-w 38822296 PMC11140989

[B100] Zhang M. Zhu L. Wu G. Liu T. Qi X. Zhang H. (2024). Food-derived dipeptidyl peptidase IV inhibitory peptides, production, identification, structure-activity relationship, and their potential role in glycemic regulation. Crit. Reviews Food Science Nutrition 64 (8), 2053–2075. 10.1080/10408398.2022.2120454 36095057

[B101] Zhang Y. Zhang Y. Chen Y. Localized GLP1 receptor pre-internalization directs asynchronous insulin and glucagon secretion. Cell metabolism (2025). 37(1),1–14. 10.1016/j.cmet.2025.06.009 PMC12276844 40664215

[B102] Zhang X. Cao C. Zheng F. Liu C. Tian X. (2025). Therapeutic potential of GLP-1 receptor agonists in diabetes and cardiovascular disease: mechanisms and clinical implications. Cardiovasc. Drugs Therapy: 1–15. 10.1007/s10557-025-07670-9 39832069 PMC12872670

[B103] Zhao L. H. Yin Y. Yang D. Liu B. Hou L. Wang X. (2016). Differential requirement of the extracellular domain in activation of class BG protein-coupled receptors. J. Biological Chemistry 291 (29), 15119–15130. 10.1074/jbc.M116.726620 27226600 PMC4946928

[B104] Zhao X. Wang M. Wen Z. Lu Z. Cui L. Fu C. (2021). GLP-1 receptor agonists, beyond their pancreatic effects. Front. Endocrinology 12, 721135. 10.3389/fendo.2021.721135 34497589 PMC8419463

[B105] Zheng Z. Zong Y. Ma Y. Tian Y. Pang Y. Zhang C. (2024). Glucagon-like peptide-1 receptor, mechanisms and advances in therapy. Signal Transduction Targeted Therapy 9 (1), 234. 10.1038/s41392-024-01931-z 39289339 PMC11408715

[B106] Zheng Z. Zong Y. Ma Y. (2025). Structural pharmacology and mechanisms of GLP-1R signaling: implications for drug design. Trends Pharmacological Sciences 46 (3), 234–250. 10.1016/j.tips.2025.03.003 40221226

[B107] Zhou Q. Zhao F. Zhang Y. Yang D. Wang M. W. (2025). Structural pharmacology and mechanisms of GLP-1R signaling. Trends Pharmacological Sciences 46, 422–436. 10.1016/j.tips.2025.03.003 40221226

[B108] Zou X. He Y. Gao Y. Wang J. Zhang J. Z. H. (2025). Understanding the activation mechanism of GLP-1R/GIPR by dual agonist tirzepatide *via* molecular dynamics and protein-peptide binding. Int. Journal Biological Macromolecules 321, 146141. 10.1016/j.ijbiomac.2025.146141 40692063

